# Histological, radiological, and clinical outcomes of sinus floor elevation using a lateral approach for pre-/post-extraction of the severely compromised maxillary molars: a study protocol for a randomized controlled trial

**DOI:** 10.1186/s13063-021-05047-5

**Published:** 2021-01-28

**Authors:** Zhaoguo Yue, Qi Liu, Haidong Zhang, Jingwen Yang, Jianxia Hou

**Affiliations:** 1 Department of Periodontology, Peking University Hospital and School of Stomatology, 22 Zhongguancun South Avenue, Haidian District, Beijing, 100081 China; 2 National Clinical Research Center for Oral Diseases, Peking University Hospital and School of Stomatology, 22 Zhongguancun South Avenue, Haidian District, Beijing, 100081 China; 3 National Engineering Laboratory for Digital and Material Technology of Stomatology, Peking University Hospital and School of Stomatology, 22 Zhongguancun South Avenue, Haidian District, Beijing, 100081 China; 4 Beijing Key Laboratory of Digital Stomatology, Peking University School and Hospital of Stomatology, 22 Zhongguancun South Avenue, Haidian District, Beijing, 100081 China; 5 BYBO Dental Hospital, Qinian Street, Dongcheng District, Beijing, 100062 China; 6 Department of Prosthetics, Peking University Hospital and School of Stomatology, 22 Zhongguancun South Avenue, Haidian District, Beijing, 100081 China

**Keywords:** Dental implants, Maxillary sinus floor elevation (MSFE), Lateral approach, Bone graft, Periodontitis, randomized controlled trial

## Abstract

**Background:**

The volume of residual alveolar bone is critical to the survival of dental implants. When the volume of alveolar bone in the posterior maxillary region is less than 4 mm, maxillary sinus floor elevation (MSFE) with the lateral approach is an effective option. Traditionally, this standard approach is usually conducted at 4–6 months after tooth extraction (standard MSFE). However, defective dentition due to extraction can impair mastication during the period of bone remodeling, especially if the molars on both sides are severely compromised and must be extracted. MSFE before extraction (modified MSFE) can take full advantage of residual tooth strength. However, the effectiveness and practicability of the modified MSFE procedure remain unknown. Therefore, the aim of this study was to compare the clinical outcomes of modified vs. standard MSFE, in order to provide references to periodontists.

**Methods/design:**

The study cohort included 25 adult patients (50 surgery sites) recruited from Peking University Hospital and School of Stomatology who met the inclusion criteria. The two sides of each patient will be randomly divided into two groups: a test group-modified MSFE or a control group-standard MSFE. The surgical duration and patient-reported outcomes (visual analog scale for discomfort) will be documented. Clinical indicators, including implant survival rates, mucosal conditions, and complications, will be recorded every 6 months during the 5-year follow-up period. The volume of the alveolar bone and marginal bone level will be assessed radiographically (cone-beam CT and periapical films) every 6 months. Histological analysis of biopsy samples retrieved from both sides will be performed to evaluate the biological features of the bone.

**Discussion:**

The current study will explore the implant survival rates, safety, reliability, effectiveness, and practicability of the modified MSFE procedure. Moreover, the extent of osteogenesis on the sinus floor will also be assessed. The results of this trial will provide strategies for the modified MSFE procedure to achieve ideal clinical outcomes.

**Trial registration:**

International Clinical Trials Registry Platform ChiCTR1900020648. Registered on 1 January 2019

**Supplementary Information:**

The online version contains supplementary material available at 10.1186/s13063-021-05047-5.

## Background

Dental implantation is a viable option to restore impaired dentition. However, an adequate volume of residual alveolar bone is critical for the survival of a dental implant [1]. For atrophied posterior maxilla with a residual bone height of less than 4 mm, it is wise to conduct maxillary sinus floor elevation (MSFE) surgery to optimize the condition of the bone prior to implantation [2].

As is generally known, MSFE can be performed via the transalveolar or lateral approach. As compared to the transalveolar approach, MSFE with the lateral approach provides a direct view of the surgical field, minimizes the risk of perforation, and ensures an adequate volume of grafted bone material [2–4]. However, multiple intra- and postoperative complications as well as discomforts caused by this procedure remain problematic [5]; therefore, various methods have been proposed to improve impaired masticatory function during the healing period [6–10]. Previous studies have mostly concentrated on methods to shorten the healing period rather than directly improve masticatory function. Therefore, the patients may experience discomfort and impaired oral function for at least 10–14 months after surgery, especially after extraction of the molars on both sides due to severe periodontitis [11].

However, it is not reasonable to deny necessary extractions due to possible partial edentulous or loss of oral function. Even with active treatment, 40% of “hopeless” teeth do not survive for longer than 4 years on average [12]. Based on this and the high success rate of dental implants, earlier strategic extraction of hopeless teeth is considered beneficial for implant placement [13]. However, the timing of extraction is very critical. As previously reported [14], it is also necessary to fully utilize and extend the capacity of the natural tooth. Given that the integrity of dentition is fundamental to high masticatory efficiency [15], optimizing the longevity of the dentition will improve the patients’ quality of life. Since extraction and MSFE are inevitable for some patients with periodontitis, it is reasonable to ask whether it is technically and clinically feasible to extract the compromised teeth after the elevation of the sinus floor.

The reliability and the safety of extended MSFE to the apical area of the neighboring teeth, as proposed by Beitlitum et al. [16], were assessed in a recent clinical trial involving 65 patients to enable future implant placement while avoiding the need for sinus reentry after extraction of the proximal teeth. It can be inferred that MSFE can be successfully achieved prior to extraction of the corresponding teeth.

Base on the dilemma of patients with severe periodontitis and the inference above, the current research proposes MSFE before extraction. In clinical practice, we try to maintain the compromised tooth to fully utilize its residual function in order to reduce the dentition-impaired period by at least 6 months. The extraction and implantation will be completed in one visit to minimize the number of surgeries. This time is a good opportunity to investigate the promoting effect of functional stimulation on bone regeneration, which may improve conditions for implantation.

### Objective and hypothesis

The major goal of the current randomized controlled trial is to compare the clinical, radiological, histological, and patient-reported outcomes of modified vs. standard MSFE.

The primary hypothesis is that modified MSFE can achieve ideal clinical outcomes similar to those of standard MSFE, while improving mastication. Considered outcomes include implant survival rate, marginal bone remodeling, and discomfort, as assessed with a visual analog scale (VAS). Histological and radiological analyses of bone quality and quantity will also be considered.

## Methods

### Overview

The proposed study is designed as a prospective single-center, split-mouth, randomized controlled trial. We plan to recruit 25 patients with severely periodontally compromised molars on both sides and in need of dental implant treatment in the atrophied posterior maxilla. The participants will be recruited by the research staff from Peking University Hospital and School of Stomatology (Beijing, China). All procedures, recalls, and analyses will be conducted at this hospital. The study protocol was approved by the Ethics Committee of Peking University Hospital and School of Stomatology (approval no. PKUSSIRB-201840191). The study has been registered with the International Clinical Trials Registry Platform (identifier no. ChiCTR1900020648).

The systemic health status of all participants will be qualified prior to treatment. All patients recruited for the study will receive standard periodontal initial therapy and periodontal maintenance at regular intervals. Once the clinical and periodontal health of the patient is confirmed, MSFE surgery will be performed. Prior to surgery, all patients will be assessed both clinically and radiographically. Initial bone height, bone quality, and alveolar width will be assessed by cone-beam computed tomography (CBCT).

### Inclusion criteria

The following are the inclusion criteria:

- Bilateral first/second molars requiring extraction due to severe periodontal disease (i.e., prognosis regarded as “hopeless” [17]) and the patient’s desire for replacement with implants
- Residual alveolar bone height of the compromised teeth ranging from 1 to 3 mm
- Age ≥ 18 years
- Healthy maxillary sinuses with intact sinus floors
- Good general health
- Non-smokers
- Signed informed consent form
- Good oral hygiene and compliance with the treatment regimen

### Exclusion criteria

The following are the exclusion criteria:

- Residual alveolar bone height of the compromised teeth more than 4 mm
- Age < 18 years
- Poor oral hygiene and uncontrolled periodontitis
- Current rhinitis, sinusitis, or a large cyst in the maxillary sinus
- Smokers
- Current and uncontrolled systemic diseases, such as diabetes mellitus, cardiovascular diseases, immune system diseases, severe osteoporosis, and/or blood disorders, such as coagulation disorders
- Long-term medication use (e.g., steroids, anti-epileptic drugs, and bisphosphonates)
- Inability or unwillingness to sign the informed consent form

### Recruitment

**Fig. 1:**
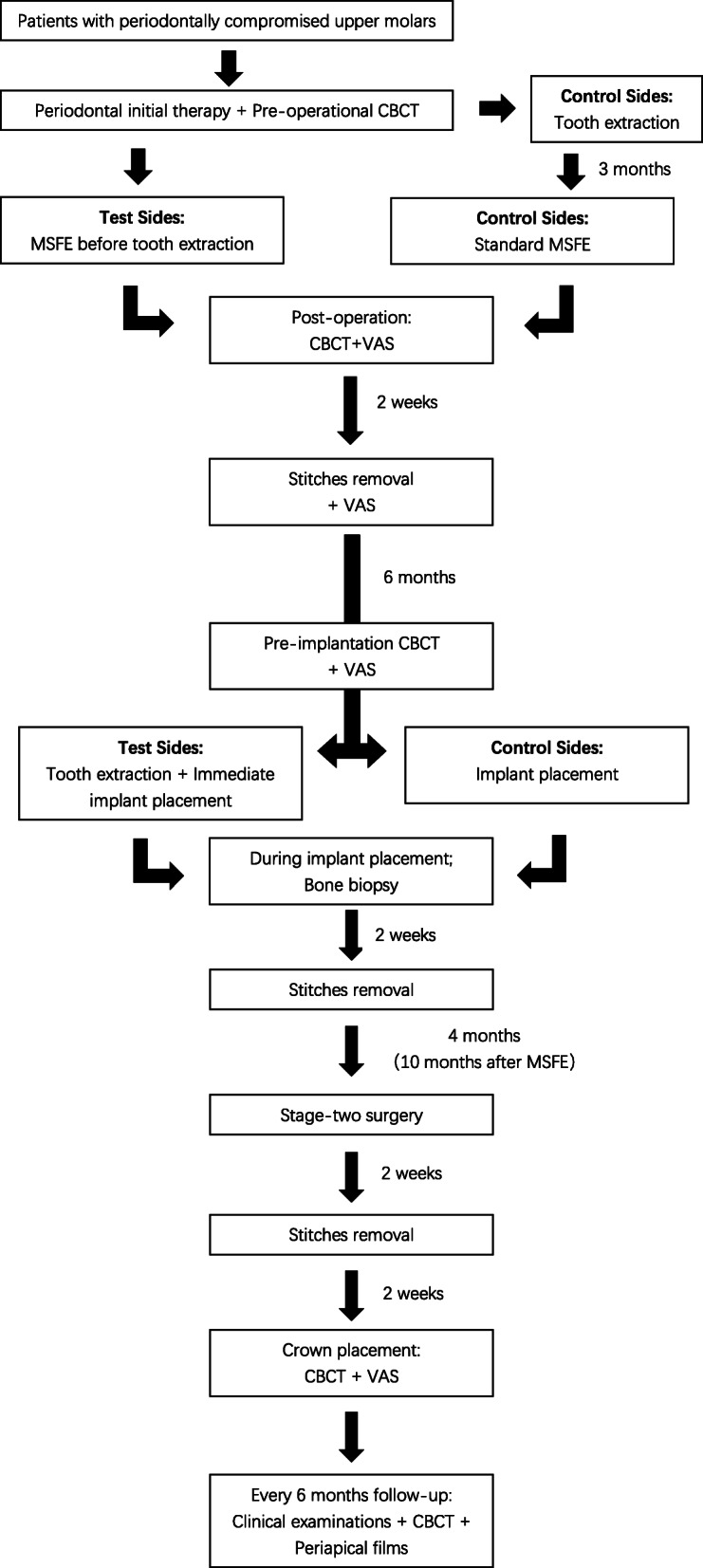
Flow chart of the treatment process

**Fig. 2:**
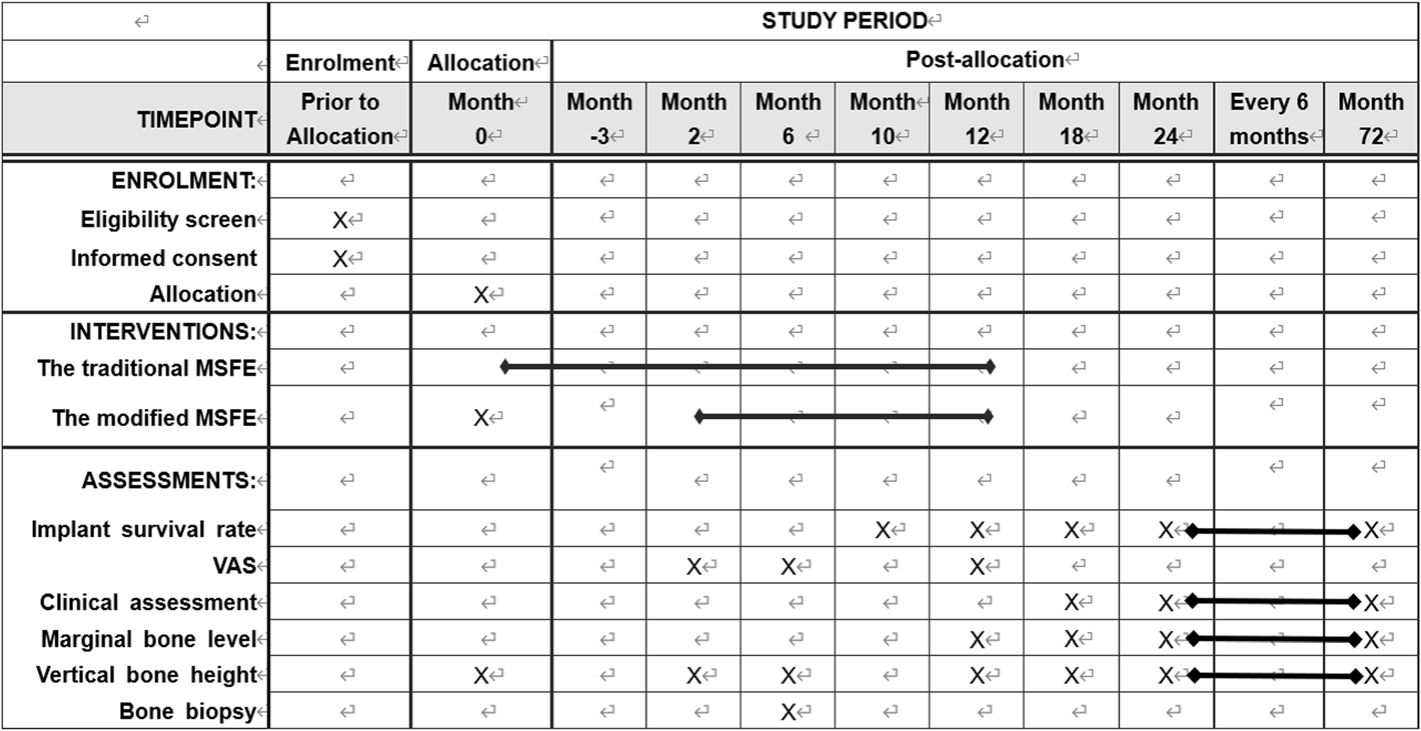
SPIRIT figure

Potential patients will be recruited from Peking University Hospital and School of Stomatology. Jianxia Hou is in charge of the local organization, while Zhaoguo Yue, Haidong Zhang, and Jingwen Yang will participate in the recruitment and ensuring consent. Eligible patients will receive a written informed consent form explaining the trial process in plain words. If interested, the study background, study methods, duty as participants, biopsy collection and storage, possible impacts on daily life, potential risks, back-up treatment plans, and rules of confidentiality will be verbally explained by the researchers to each of the study participants. The study participants are allowed to ask any question related to the trial and have the right to be informed of all the details of the study. After we are certain that each patient fully understands the goals of the trial, an informed consent form must be personally signed prior to study inclusion. A flow chart of the treatment process is presented in Fig. 1. A SPIRIT (Standard Protocol Items: Recommendations for Interventional Trials) figure is presented in Fig. 2.

### Allocation, randomization, and blinding

An external researcher blinded to the operations and analysis process will perform the randomization using the Randomizer for Clinical Trials web-based randomization service (Institute for Medical Informatics, Statistics and Documentation, Medical University of Graz, Graz, Austria). Each patient will be assigned a number within a corresponding envelope sealed by the external researcher and allocated to one of two groups: the test group (MSFE before tooth extraction) or the control group (MSFE after tooth extraction).

In accordance with the principle of blindness, the surgeon will have no access to the statistical analysis process. Similarly, the statistical analyst will be blinded to the interventions and groupings.

### Surgical interventions

#### Implant and bone substitute

All patients will receive Straumann BL implants with sand-blasted, large grit, acid-etched implant surfaces (Institut Straumann AG, Basel, Switzerland). Deproteinized Bovine Bone Mineral (Bio-oss®; Geistlich Pharma AG, Wolhusen, Switzerland) will be used as a bone substitute.

### MSFE

For the test group, with the exception of a few specific changes, the surgical procedure will largely mirror the method reported by Tatum [18]. The sinus elevation procedure will be performed before the extraction of the compromised tooth/teeth. Prior to surgery, the compromised teeth will be treated to eliminate inflammation of the periodontal tissue. Once the periodontal status of the compromised teeth is deemed clinically healthy (plaque index ≤ 1; no further bleeding on probing; probe depth≤ 4 mm), a crevicular incision will be made, which is designed to be extended to at least one adjacent tooth both mesially and distally. A full-thickness access flap will be prepared, and an access window (5 × 8 mm) will be created on the lateral wall of the maxillary sinus with the use of a round diamond bur under irrigation with sterile saline. The lower border of the window will be at least 3–5 mm above the floor to maintain an appropriate distance from the teeth on the site. The sinus membrane will be raised by at least 10 mm, exceeding the apical range of the tooth (both buccolingually and mesiodistally). The generated cavity within the maxillary sinus will be filled with deproteinized bovine bone mineral (Bio-Oss®). After the bone substitute is placed, an absorbable collagen membrane (Bio-Gide®; Geistlich Pharma AG) will be placed to cover the antrostomy defect. Interrupted or mattress PROLENE monofilament non-absorbable sutures (Ethicon, Inc., Johnson & Johnson International, Bridgewater, NJ, USA) will be placed to close the primary flap. All patients will receive preoperative antibiotic prophylaxis, consisting of 500 mg of amoxicillin three times daily for 7 days postoperatively. After a healing period of 6 months (prior to dental implant placement), CBCT will be performed to assess vertical bone height at the planned dental implant sites.

For the control group, the procedure will be performed 3 months after the extraction of the compromised teeth/tooth. Besides, standard MSFE will be conducted in accordance with the method described by Tatum [18].

### Implant placement

Six months after the MSFE procedure, dental implant surgery will be performed under local anesthesia. For the test group, the compromised teeth will be extracted with minimal invasion and biopsied with a hollow trephine drill (internal diameter, 2 mm; length, 6 mm) and the sample will be copiously irrigated with sterile saline before fixated in phosphate-buffered formaldehyde. The implant site will be prepared simultaneously, and the position and orientation will be restoratively driven. Straumann dental implants (diameter, 4.8 mm; length, 10 mm) will be inserted into the planned site, at an adequate depth so that the platform is 1 mm below the buccal plate. Then, the implant will be mounted with healing caps and sutured with PROLENE monofilament non-absorbable sutures (Ethicon, Inc.). To double-check the implant position, postoperative periapical radiographs will be obtained with the paralleling technique.

For the control group, a crestal incision will be made with mesial and distal buccal vertical release incisions. A full-thickness mucoperiosteal flap will be raised to expose the alveolar ridge and grafting site. Other steps of the procedure will be performed as described above.

For both groups, after 4 months of healing of the unloaded implants, a two-stage surgery will be performed under local anesthesia to replace the implant cover screws with healing abutments. After an additional month, the crowns will be placed.

### Outcomes

#### Baseline assessment

The surgical duration of the MSFE procedure will be calculated from the time of the initial incision to the end of wound closure. CBCT will be performed before and immediately after the MSFE procedure. Periapical images will be obtained as soon as the crowns are placed. These digital radiographic data will be used as the baseline radiographic data. Patients will be asked to score their discomfort (i.e., pain and edema levels) immediately and 2 weeks after surgery using a visual analog scale (VAS). The condition of the mucosa will be assessed at 6 months as the baseline clinical outcome.

#### Bone biopsy and histological/histomorphometric analyses

After an osteogenesis period of 6 months, histological and histomorphometric analyses will be performed of biopsy samples retrieved from the grafting sites. The bone biopsy samples will be collected during dental implant surgery with the use of a hollow trephine drill, fixated in 10% formaldehyde (pH 7.4; 4 °C), transferred to 70% ethanol, and stored until used for histomorphometric analysis. The samples will be subsequently dehydrated in descending concentrations of ethanol, embedded in low temperature polymerizing methyl methacrylate, sectioned (thickness, 4 μm), and stained with both hematoxylin-eosin and modified Mallory aniline blue. The sections will be divided into regions of interest (ROI). For each ROI, histomorphometric measurements will be performed blindly. The bone volume, graft volume, osteoid volume, and connective tissue volume will be calculated as a percentage of the total tissue volume. The osteoid-graft perimeter and connective tissue-graft perimeter will be calculated as a percentage of the total graft perimeter [19].

#### Follow-up assessment

All study participants will undergo CBCT at 6 (immediately before implant placement) and 12 months (immediately before crown placement) after the MSFE procedure to assess the outcome. A VAS will be distributed to each participant at 6 and 12 months after surgery to evaluate the oral function over the whole restoration period.

Six months after implant placement (12 months after MSFE), crowns will be placed, which will be designated as the starting point of the 5-year follow-up period. Patients will be called back for re-visits at 18, 24, 30, 36, 42, 48, 54, 60, 66, and 72 months after MSFE to document the implant survival rate, mucosa conditions, and complications. Periapical radiographs will be obtained to survey marginal bone levels, and CBCT analysis will be conducted to observe changes in alveolar bone volume during every visit.

#### Primary outcomes of the trial

The primary parameter of the current trial is implant survival rate, which will be calculated as the proportion of retained implants at 5 years of follow-up.

The criteria of survival or failure of an implant were referred to the consensus of the International Congress of Oral Implantologists [20]. The implant will be regarded as failed if any of the following happens: (a) pain on function, (b) mobility, (c) radiographic bone loss > 1/2 length of the implant, (d) uncontrolled exudate, and (e) no longer in the mouth. Otherwise, the implant will be regarded as surviving.

#### Secondary parameters

The secondary parameters of this study include the following:

1. The marginal bone level: The marginal bone level will be observed on periapical images obtained with the paralleling technique. Measurements of the mesial and distal bone crest levels adjacent to each implant will be made to the nearest 0.01 mm. The distance between the coronal margin of the implant collar and the most coronal point of the bone-to-implant contact will be recorded. If the margin is above the implant-abutment junction, the distance between the collar and the highest bone level will be recorded. Implants with bone up to the coronal margin of the implant collar will be given a score of zero. The average mesial and distal measurements will be calculated to indicate the stability of the marginal bone [21].
2. The alveolar bone height: The measurements of bone height will be performed by CBCT analysis. CBCT analysis will be conducted using the Mimics Three-dimensional Medical Image Processing Software (version 18.0; Materialise, Leuven, Belgium). Bone height at the implantation site will be measured at three points on the sagittal plane (the middle of insert placement, 2 mm mesial and 2 mm distal). The average of the measurements at these three points will be regarded as the volume of alveolar bone.
3. The surgical duration of the MSFE procedure, which will be calculated from the time of the initial incision to the end of wound closure.
4. Complications during and after the surgery, including infection, hematoma, nasal bleeding, benign paroxysmal positional vertigo, hemorrhage, Schneider’s membrane perforation, infection (especially at the grafting site), sinusitis, and other potential risks that cannot be anticipated before the intervention.
5. Patient-reported outcomes (VAS): Intra- and postoperative discomfort (pain and edema) as well as restoration period discomfort (oral function) will be assessed by applying a VAS. Patients will be asked to rate intraoperative discomfort and postoperative pain levels using a 100-mm scale with “very dissatisfied” on the left and “very satisfied” on the right [22, 23].
6. Histological evaluation of the biopsy sample from the implant site: Histological evaluation will be conducted as previously explained. For each biopsy, the bone volume, graft volume, osteoid volume, connective tissue volume, osteoid-graft perimeter, connective tissue-graft perimeter, and graft perimeter will be calculated.
7. The condition of the mucosa around the implant will be assessed according to the following parameters: probing depth, sulcus bleeding (bleeding index), plaque around the restorations (modified plaque index), and keratinized mucosa width. The occurrence of biological complications (peri-implantitis and peri-implant mucositis) will be recorded.

### Sample size

The PASS sample size software (version 11.0; NCSS, LLC, East Kaysville, UT, USA) was used to estimate the sample size needed for the current study based on the following formula: *N* = (1/Q1 + 1/Q2) [(*Z*_*α*/2_ + *Z*_*β*_)*σ*/*δ*]^2^. As the primary parameter, the implant survival rate was used to estimate the sample size. The mean 5-year survival rate and standard error (SE) were calculated as described in a previous study [21] (survival rate after 5 years = 97.8%; mean of paired difference [*δ*] = 5.0%; SE [*σ*] = 5.0%) with significance criteria of *α* = 0.05 (error) and *β* = 0.10 (type II error). A two-tailed, non-inferiority analysis was performed. To reach a power of 90%, at least 16 patients (32 sites) should be included. Assuming a dropout rate of 20%, 20 patients (40 sites) were needed. Considering the cluster effect caused by the split-mouth design, we further enlarged the sample size to 25 patients (50 sites) for this trial.

### Data collection and management

All data will be acquired only by the members of the investigative team who are obligated to keep the data confidential from the public and only use the data for scientific research. The data will be documented in an exclusive computer as well as on paper. The data will be uploaded to an online data management team (ResMan® research manager repository, http://www.medresman.org) who will also oversee the trial process. To ensure confidentiality, user names and passwords will be assigned to the investigators. Two experimenters will independently conduct the statistical analyses. If there are more than one implant placed on one side, to avoid potential bias, only one random implant will be evaluated.

### Training and calibration

One expert (Qi Liu) with more than 10 years of working experience will be appointed to conduct the MSFE procedure. He will also receive training with the modified MSFE procedure with the use of a dental simulation model to fully master the novel surgical procedure prior to meeting with the first patient.

Before the examination, two independent investigators, other than the operator, will perform the radiographic measurements and histological evaluations using instructional CBCT data and histological sections. Both investigators are medical practitioners with at least 3 years of working experience and will be trained to adequate levels of accuracy and reproducibility to guarantee conformity. Clinical parameters, VAS scores, complications, and surgical durations will be collected by one researcher with at least 3 years of working experience to assure consistency.

### Statistical analysis

For continuous data, such as clinical parameters, consistency between the two examiners will be assessed with the intra-class correlation coefficient. For descriptive data, consistency will be assessed using Cohen’s *k* statistic. Descriptive statistics include frequency values (absolute and relative values) and metric data (arithmetic mean, standard deviation, and median). The measured data will be expressed as the mean ± standard deviation or median (quartile spacing) and the enumerated data as percentages. Cox regression and Kaplan-Meier survival analysis will be conducted to evaluate the survival rate of implants. The incidence of complications will be compared using the *χ*^2^ test. The paired-samples *t* test will be applied to evaluate the VAS scores, clinical parameters, degree of marginal bone remodeling, and vertical bone gain of the test and control groups. Histomorphometric measurements of the biopsies of both groups will be compared using the non-parametric Wilcoxon test for paired samples. All statistical analyses will be performed using IBM SPSS Statistics for Windows, version 19.0. (IBM Corporation, Armonk, NY, USA). Two-sided probability (*p*) values of < 0.05 will be considered statistically significant.

### Missing data

When estimating the study’s sample size, the possibility of loss to follow-up will be considered and factored into the calculation. Moreover, other missing data will be accounted for by handling drop-outs as non-success or non-survival, in accordance with the intention-to-treat principle.

### Ethical considerations

#### Ethical approval

The study protocol was approved by the Ethics Committee of Peking University Hospital and School of Stomatology (approval no. PKUSSIRB-201840191). Eligible patients will receive information regarding the study and consent forms. Patients who are unwilling to sign the consent will be excluded from the analysis.

### Withdrawal

Prior to any intervention, each patient will be fully informed about the goals and risks of the current study. All participants have the right to withdraw from the study at any time for any reason. All patients will receive required treatment regardless of the study participation.

### Dissemination of results

The study outcomes will be recorded and published in an international peer-reviewed journal. For public access, analysis of the study outcomes will be uploaded to Chictr.org.cn.

### Oversight

#### Trial management

The current clinical trial is coordinated by the National Clinical Research Center for Oral Diseases (Beijing, China). This coordinating center will assist the research team and provide support in many areas including trial design, study management, quality assurance, data analysis, dissemination and trial close-down, etc.

### Steering committee

In order to steer and ensure the process of the trial, several patients and public representatives, as well as three independent clinicians and a statistician, were appointed as members of the trial steering committee. The committee members are not involved in performing the trial and will meet with the research group every 3 months. The responsibilities of the trial steering committee include making recommendations to the trial research group regarding the conduct of the trial, recruitment and follow-up processes, data management and monitoring, statistical analysis of outcomes, and to assess the rate of progress to ensure that the trial is conducted in accordance with the study plan.

### Data monitoring

Progression of the trial, adverse events, and data quality will be monitored by an independent Data (and Safety) Monitoring Committee (DMC) consisting of members of the Ethics Committee of Peking University Hospital and School of Stomatology, who are independent of the trial investigators, research team, and sponsors. An inspector appointed by the DMC will meet with the research team regularly (every 3 months) to monitor the progress of the study and conduct interim analyses. Additionally, the DMC will retain the right to terminate the trial if harms or risks emerge according to the interim results.

### Harms

There is no anticipated harm to the study participants, and there is a low risk for the MSFE procedure to fail. All surgeries will be conducted by the same experienced dentist (QL), and all necessary measures will be taken to minimize the risk of harm to the study participants. If the surgery fails, the patient will be provided with a back-up restoration plan, such as a removable partial denture. Surgical failure together with any unintended adverse effect or serious adverse event will be immediately reported to the DMC.

### Audits

An inspector appointed by the DMC will review the incoming data every 3 months independently from the investigators and sponsors. The inspector will review whether each electronic case report form is completed accurately. All discrepancies in the electronic case report form will be corrected by the principal investigator.

## Discussion

As previously reported [16], MSFE before tooth extraction is both safe and reliable. However, the effectiveness of this procedure to reduce discomfort remains uncertain, especially when both sides of molars are periodontally compromised and must be extracted.

In order to reduce the number of procedures, implantation is performed immediately after the MSFE procedure. At the same time, maintaining the tooth will minimize discomfort caused by defective oral function for a period of 3–4 months. We propose that this new clinical strategy will improve patient satisfaction with the procedure.

Because the loss of available bone volume is caused by resorption of the alveolar bone and pneumatization of the sinus mainly due to the loss of functional stimulation of the teeth [24], we presumed that functional stimuli will be beneficial to the outcomes of floor augmentation, both radiographically and histologically. The extent of osteogenesis will also be compared to determine whether the hypotheses will be accepted.

The results of the present study will determine if the modified MSFE procedure, including indications, detailed methods, postoperational complications, and management, is actually beneficial.

### Challenges

Because we plan to elevate the Schneider’s membrane with the tooth/teeth on site, it will be more difficult to keep the membrane intact during the lifting process, especially if the sinus floor is proximal to the root apex and, thus, full of “lumps.” Evaluations, including CBCT analysis, should be carefully conducted before the procedure. Operators should be skilled, so that the membrane can be totally stripped along the contour of the floor. Fixing methods should be prepared beforehand as a back-up plan in case of perforation.

### Trial status

This trial has been registered at Chictr.org.cn and recruitment for the study is ongoing. This is the fifth revision of the study protocol (2020-8-10). Recruitment began on February 1, 2019, and is expected to be completed by February 1, 2021.

## Supplementary Information

**Additional file 1.** 2013 SPIRIT Checklist.

**Additional file 2.** Record of protocol amendments.

## Data Availability

The datasets generated and analyzed during the current study are available from the ResMan® research manager repository (http://www.medresman.org).

## Abbreviations

CBCT: Cone-beam computed tomography
CT: Computed tomography
MSFE: Maxillary sinus floor elevation
ROI: Region of interest
VAS: Visual analog scale
