# Supplementary material for: Histological, radiological, and clinical outcomes of sinus floor elevation using a lateral approach for pre-/post-extraction of the severely compromised maxillary molars: a study protocol for a randomized controlled trial
Source: Trials. 2021 Jan 28;22:101. doi: 10.1186/s13063-021-05047-5 (PMC7844904; doi:10.1186/s13063-021-05047-5)
Supplement: Supplementary file 2 — Additional file 2. Record of protocol amendments. [file 13063_2021_5047_MOESM2_ESM.docx]

**Protocol Amendments Record**

| No. | Ver. | Amendment Date | Reviser/Composer | Sections Being Amended |
| --- | --- | --- | --- | --- |
| 1 | 1 | 2018-10-22 | Zhaoguo Yue, Jingwen Yang and Jianxia Hou | Draft Composition |
| 2 | 2 | 2018-12-06 | Zhaoguo Yue and Jianxia Hou | Background, Outcomes and Discussion |
| 3 | 3 | 2019-4-15 | Zhaoguo Yue, Jingwen Yang, Yalin Zhan and Jianxia Hou | Primary parameters and Challenges |
| 4 | 4 | 2020-4-10 | Zhaoguo Yue and Jianxia Hou | Sample size & Training and calibration |
| 5 | 5 | 2020-7-19 | Zhaoguo Yue and Jianxia Hou | （Major revision） |
| 6 | 6 | 2021-01-03 | Zhaoguo Yue and Jianxia Hou | （Minor revision） |
